# The Scars of Childhood Adversity: Minor Stress Sensitivity and Depressive Symptoms in Remitted Recurrently Depressed Adult Patients

**DOI:** 10.1371/journal.pone.0111711

**Published:** 2014-11-13

**Authors:** Gemma Kok, Gerard van Rijsbergen, Huibert Burger, Hermien Elgersma, Heleen Riper, Pim Cuijpers, Jack Dekker, Filip Smit, Claudi Bockting

**Affiliations:** 1 Department of Clinical Psychology, University of Groningen, Groningen, The Netherlands; 2 Department of General Practice, University of Groningen, University Medical Center Groningen, Groningen, The Netherlands; 3 Interdisciplinary Center for Psychiatric Epidemiology, University of Groningen, University Medical Center Groningen, Groningen, The Netherlands; 4 Department of Clinical Psychology and EMGO + Institute for Health and Care Research, VU University and VU University Medical Centre, Amsterdam, the Netherlands; 5 Leuphana University, Lüneburg, Germany; 6 Research Department, Arkin Mental Health Institute, Amsterdam, The Netherlands; 7 Department of Clinical Psychology, VU University, Amsterdam, The Netherlands; 8 Department of Epidemiology and Biostatistics, EMGO + Institute for Health and Care Research, VU University Medical Centre, Amsterdam, the Netherlands; 9 Trimbos Institute (Netherlands Institute of Mental Health and Addiction), Utrecht, the Netherlands; National Center of Neurology and Psychiatry, Japan

## Abstract

**Background:**

Childhood adversity may lead to depressive relapse through its long-lasting influence on stress sensitivity. In line with the stress sensitization hypothesis, minor (daily) stress is associated with depressive relapse. Therefore, we examine the impact of childhood adversity on daily stress and its predictive value on prospectively assessed depressive symptoms in recurrently depressed patients.

**Method:**

Daily stress was assessed in recurrently depressed adult patients, enrolled into two randomized trials while remitted. The reported intensity and frequency of dependent and independent daily stress was assessed at baseline. Independent stress is externally generated, for example an accident happening to a friend, while dependent stress is internally generated, for example getting into a fight with a neighbor. Hierarchical regression analyses were performed with childhood adversity, independent and dependent daily stress as predictor variables of prospectively measured depressive symptoms after three months of follow-up (n = 138).

**Results:**

We found that childhood adversity was not significantly associated with a higher frequency and intensity of daily stress. The intensity of both independent and dependent daily stress was predictive of depressive symptom levels at follow-up (unadjusted models respectively: B = 0.47, t = 2.05, *p* = 0.041, 95% CI = 0.02–0.92; B = 0.29, t = 2.20, *p* = 0.028, 95% CI = 0.03–0.55). No associations were found between childhood adversity and depressive symptoms at follow-up.

**Conclusion:**

No evidence was found supporting stress sensitization due to the experience of childhood adversity in this recurrently depressed but remitted patient group. Nevertheless, our research indicates that daily stress might be a target for preventive treatment.

**Trial Registration:**

Trial A: Nederlands Trial Register NTR1907 Trial B: Nederlands Trial Register NTR2503

## Introduction

Major Depressive Disorder (MDD) is a highly recurrent disease with reported relapse and recurrence rates that range from 50–90% [1], [2]. Each depressive episode heightens the risk of additional relapses and recurrences [1], [3]–[5]. For readability we refer to the more conservative term relapse in case of relapse and recurrence [6]. There is ample evidence that the experience of childhood adversity is related to the persistence of depression and depressive relapse, even after successful treatment [7]–[9]. More specifically, sexual abuse and emotional neglect seem to be associated with a poor prognosis of depression, and appear as independent determinants of chronicity of the disorder [10]–[13]. Knowledge on how childhood adversity leads to a poor prognosis of depression would provide insight into the causal processes and might help tailor treatment.

### Vulnerability-stress

An explanation of childhood adversity influencing the prognosis of depression, might be found in vulnerability-stress models [14]–[17]. Vulnerabilities, such as exposure to childhood adversity, are suggested to predispose someone to develop psychopathology after stress. Possibly, vulnerabilities cause heightened sensitivity to stress on the long-term. Indeed associations are found between childhood adversities and an increased sensitivity to daily stressors in adult life [18]–[20]. This is supported by neurobiological studies as well, where a link between childhood adversities and epigenetic modifications to sensitivity to stress has been demonstrated [21].

#### Stress sensitivity

Stress, such as life events or daily stressors, is known as one of the most consistent predictors of respectively onset and relapse in MDD [22]–[24]. According to the *stress sensitivity* or *kindling* hypothesis [25], with increasing numbers of previous depressive episodes, the role of major life stress diminishes and minor life stress is considered as a more important predictor of depressive relapse [22], [26], [27]. There is indeed evidence that minor life stress, such as the loss of personal belongings, plays a role in initiating the return of depressive symptoms and depressive episodes [22], [23], [28]–[30]. So far, of the three studies that examined the impact of childhood adversities on stress and subsequent depression, two demonstrated that the experience of childhood adversity was associated with higher sensitivity to stress, which successively heightened the risk of depression onset in adulthood [31], [32]. Conversely, the third study found that childhood adversity did not lead to increases in stress in adulthood and therefore depression in an elderly cohort (55+n = 1887) [18]. Apart from the mere exposure to stress, McLaughlin and colleagues [31] demonstrated that exposure to childhood adversity was related to a higher perceived intensity of adult daily stress. They suggest that a higher perceived intensity of stress could lead to negative mental health consequences after stress exposure.

#### Stress generation

According to the potentially modifiable *stress generation* hypothesis of Hammen [33], characteristics and behaviors of persons themselves may lead to higher generation of stress. In this hypothesis, stress influenced by a person, such as having a disagreement, is defined as dependent stress and is considered to be more related to depression than externally generated independent stress, such as the loss of a friend (i.e. stress generation; [33]. In a recent study by Liu and colleagues [34] the experience of childhood emotional neglect came forward as a unique predictor of dependent stress generation in participants with a history of depression, while independent stress was not a predictor. Depression itself is related to a heightened stress generation, even during remission [33], [35]. The influence of childhood adversity on stress could therefore eventually be overtaken by the influence of depression itself. Whether childhood adversity is associated to stress, irrespective of previous episodes, is unknown.

### Current study

In the current study we examine if an association between childhood adversity and the return of depressive symptoms exists in a currently remitted but recurrently depressed patient sample and if this is mediated by daily stress. We first studied whether childhood adversity was related to 1) the return of depressive symptoms assessed at three month follow-up, and 2) whether this relationship was partially mediated by the reported frequency and intensity of dependent and independent daily stress. Finally, additionally and aside from the mediation analysis, we examined 3) whether the reported frequency and intensity of dependent and independent daily stress, was predictive of depressive symptoms at follow-up in recurrently depressed remitted patients. In line with Monroe et al. [30], dependent and independent daily stress were examined separately to test for the *stress generation* hypothesis. We expected only dependent stress to be predictive of depressive symptoms after remission.

## Method

The protocol for these trials and supporting CONSORT checklist are available as supporting information; see Checklist S1 and Protocol S1 (trial B) and Protocol S2 (trial A).

### Participants

The total sample consisted of 309 recurrently depressed patients that entered the study while remitted. They were recruited as part of two Randomized Controlled Trials (RCT) evaluating the effectiveness of Preventive Cognitive Therapy (PCT) to prevent relapse in depression. Trial A included remitted recurrently depressed patients that used antidepressants (n = 112), recruited from July 2009 till August 2012, and examines the effectiveness of face to face PCT in addition to or as alternative for antidepressant medication (ADM) versus ADM alone [36]. Trial B included remitted recurrently depressed patients (n = 197), recruited from March 2010 till August 2012, to examine the effectiveness of an online version of PCT in addition to Treatment as Usual (TAU) versus TAU alone [37]. There were no restrictions regarding the type or frequency of current TAU and TAU could consist of ADM treatment, primary care, secondary care or no treatment at all.

All participants were currently in remission at study start, for minimally two months but no longer than two years and experienced at least two depressive episodes in the past, assessed by the Structured Clinical Interview based on the Diagnostic and Statistical Manual of Mental Disorders (SCID-I; DSM-IV) [38], and a score of 10 or below on the 17-item Hamilton Rating Scale for Depression (HRSD_17_) [39]. The SCID-I, administered by trained researchers over the telephone, was used to assess the number of previous Major Depressive Episodes (MDE), their timing and duration. The two most recent episodes of depression were assessed at symptom level in the SCID-I interview, in which the severity of an episode was established by assigning severity scores based on the number of symptoms (5 symptoms corresponds to mild, 6–7 symptoms corresponds to moderate, whereas 8–9 symptoms corresponds to severe depression). All other episodes were assessed by the core DSM-IV-TR criteria depressed mood (A1), or loss of interest (A2). The SCID-I was also used to exclude participants with: a) current or past mania or hypomania, b) current or past psychosis, c) current alcohol- or drug abuse, d) predominant anxiety disorder. Further exclusion criteria were recent electroconvulsive therapy and organic brain damage. All baseline data were acquired using online questionnaires before PCT took place. Both studies were approved by the Medical Ethical Committee and the participants provided written informed consent. All participants were above 18 years of age and able to consent. The authors confirm that all ongoing and related trials for this intervention are registered.

### Measures

#### Childhood adversities

Adversities before the age of 16 were assessed retrospectively by the Dutch version of the Life Events Questionnaire (LEQ) [40]. Previous research rated the predictive validity of the LEQ as good [40]. Emotional neglect and emotional abuse were not assessed by the LEQ. We assessed information with questions 5a, 12 and 13, respectively concerning the occurrence of a) death of a parent, b) being the victim of sexual abuse or, c) being the victim of physical abuse. Questions could be answered with yes (score  = 1) or no (score  = 0). Although there is a variety of adverse events, many studies in this field focus on these specific events, which is why these were selected [12], [13], [41]–[43]. A dichotomous variable was made, in which the presence of one or more of the adversities assessed with questions 5a, 12, and 13 were coded as 1, and the absence of all three was coded as 0.

#### Daily stress

The Dutch version of the Everyday Problem Checklist was used to assess the occurrence of 114 daily stressors in the three months preceding the baseline measurement (EPCL) [44]. The items were assigned to the subscales dependent stress (28 items) or independent stress (21 items) based on the manual of the EPCL [44]. A subdivision into these subscales is useful because dependent stress is said to be more related to recurrent depression [45]. An example of a dependent event is *you got into a conflict with a colleague*, and an independent event is *you had to wait long at an appointment*. Additionally, the subscales total frequency and total intensity of dependent and independent stress were calculated in accordance to the manual of the EPCL. The intensity of daily stress describes how the impact of stressors is experienced and the score could range from 0 (no impact) to 3 (very much impact). The reliability of all the 114 EPCL items was α = .97, this was α = .79 for the dependent subscale (28 items) and α = .71 for the independent subscale (21 items).

#### Depressive symptoms

The Dutch translation of the Inventory of Depressive Symptomatology was used to measure depressive symptoms at baseline and three month follow-up (IDS-SR) [46]. The inventory contains 30 items which can be answered on a 4-point scale, ranging from 0 (no symptom) to 3 (almost always troubled by symptom). The overall score is calculated by adding up all scores and ranges from 0–90. A score of 0–13 is categorized as no symptoms, 14–25 as mild symptoms, 26–38 moderate symptoms, 39–48 as severe symptoms and above 49 as very severe symptoms. The reliability of this measure according to Rush et al. [46] was good (α. = .79–.85). In this study the reliability was good as well (α = .77).

### Statistical analyses

All analyses were performed using SPSS version 20.0 and we considered two-sided *p*-values <.05 to be statistically significant. The characteristics of the study populations of the two trials were compared. Chi square tests were used to test differences in dichotomous variables and independent sample T-Tests were applied to normally-distributed continuous variables, for non-normally distributed variables the non-parametric Mann-Whitney U statistic was used.

In order to account for missing data on depressive symptoms, prospectively measured at three month follow-up, we used multiple imputation by chained equations. Multiple imputation is a state-of-the art technique, because it reduces the chance of systematic bias due to non-random missing data [47]. Forty imputations were performed and were combined according to Rubin's rules [48]. We restricted the analyses to the group that was randomized to the control conditions of both trials (continuation of ADM and TAU), because the experimental treatment (PCT) could have interacted with the effect of childhood adversity or daily stress on depressive symptoms. Separate linear regression analyses were performed with either childhood adversity or daily stress included as the only independent variable in the first step of the model. In step two we adjusted for gender, treatment group (TAU or continuation of ADM) and in step three for the number of previous depressive episodes.

Female gender was adjusted for because it is positively associated with both stress and depression and is no intermediary variable [49]. The number of previous episodes of depression is one of the most important predictors of future depression [1] and could be considered a confounder. However, previous episodes may be part of the causal chain between childhood adversity and present depression and including previous episodes could imply overcorrection. Therefore, this variable was only included in the final supplementary step to investigate whether adversity was related to current depression independent of previous episodes. We were interested in the association between daily stress and depressive symptom levels at follow-up. Therefore, we did not control for baseline depressive symptoms, or equivalently partial them out, because this would result in examining the extent to which daily stress predicts future depressive symptoms on top of current depressive symptoms, i.e. daily stress as a predictor of three month change in depressive symptoms.

If an overall association was found between childhood adversity and depression [50], we performed another regression analysis with presence of childhood adversity entered in the first step and daily stress in the second step to examine if daily stress was a mediator of the possible relation between childhood adversity and depressive symptoms. The relative change in the regression coefficient for childhood adversity when daily stress was added as an independent variable was assumed to be a measure of mediation and was expressed as a percentage. The regression analyses were performed separately for dependent and independent daily stress and also for the daily stress intensity and frequency. Not all daily life stressors are related to poor depression outcomes, and differentiating between the broad category of daily life stress is advised [30]. Therefore, the four daily stress subscales were treated as four distinct categories, as we expected them to be differentially related to depressive symptoms.

## Results

**Figure 1:**
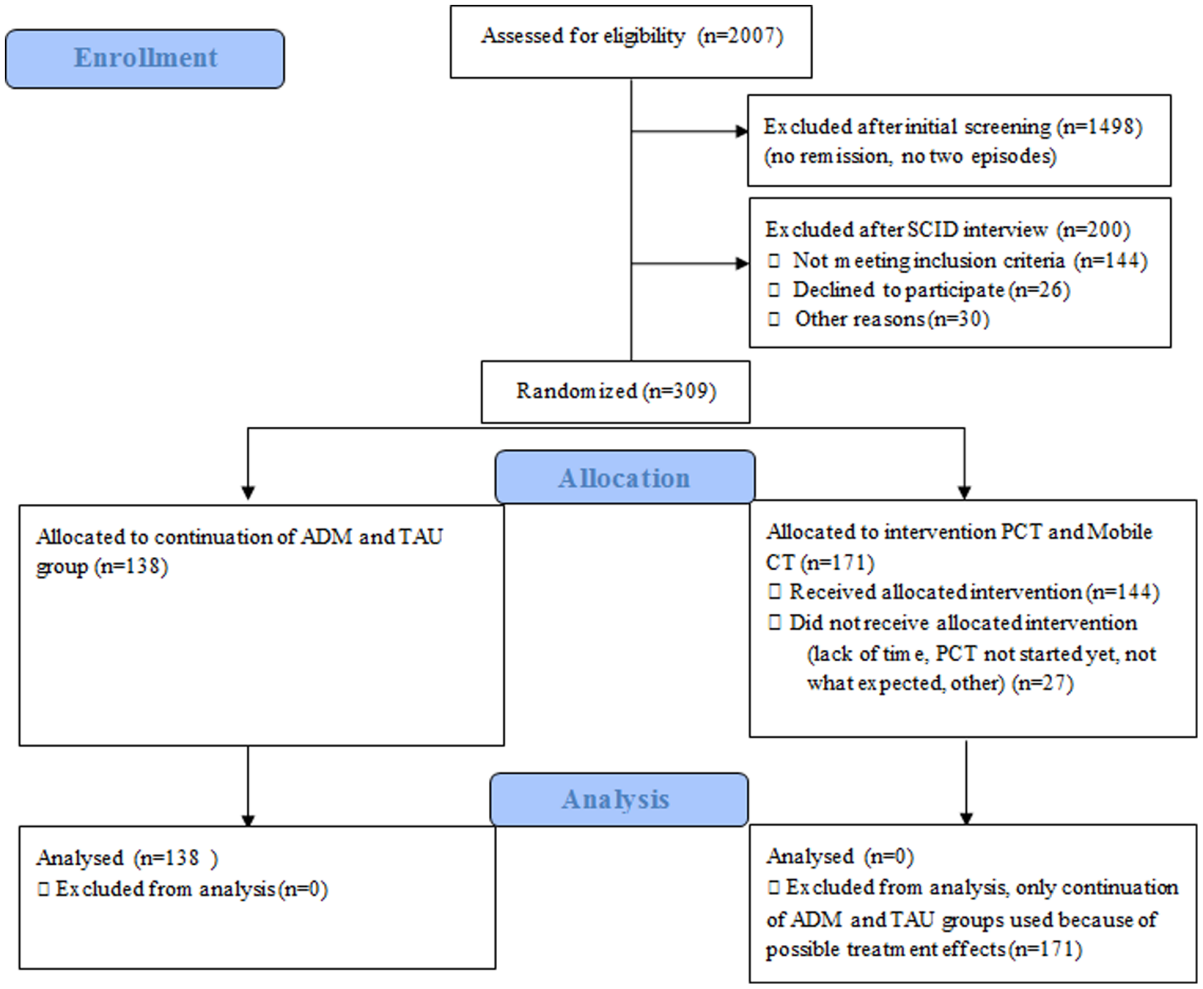
Consort Flow Diagram of participant flow.

Figure 1 provides an overview of the number of all the participants that were assessed and randomized to the trial conditions. Of the total group, n = 138 participants were assigned to the TAU group (n = 82, Trial B) and continuation of ADM group (n = 56, Trial A), of which n = 36 (26%) participants suffered at least one childhood adversity. Table 1 shows that most participants were female (68.1%) and had a mean age of 48.16 years (SD  = 10.4). Participants experienced a median of four previous depressive episodes (IQR  = 3.0). The baseline level of depressive symptoms was low, with a mean score of 3.53 (SD  = 2.8) on the HDRS_17_ [39], and a mean of 17.61 (SD  = 10.2) on the IDS-SR_30_ [46]. The trials did not differ on any of the demographic and clinical characteristics, except for ADM use (*p*<.001).

**Table 1:** Baseline demographic and clinical characteristics (n = 138).

Variable	Total (N = 138)	Trial A (N = 56)	Trial B (N = 82)	p^a^
Age, mean (SD)	48.16 (10.4) (n = 138)	48.32 (10.1) (n = 56)	48.05 (10.7) (n = 82)	.526
Female gender, no. (%)	92/138 (68.1)	35/56 (62.5)	57/82 (68.3)	.391
ADM at recruitment, no. (%)				
Yes ADM	102/138 (73.9)	56/56 (100.0)	46/82 (56.1)	.000
Current psychotherapy	47/138 (34.1)	20/56 (35.7)	27/82 (32.9)	.680
Age of first MDD episode, mean (SD)	30.10 (13.4) (n = 134)	30.27 (13.1) (n = 52)	29.79 (13.9) (n = 82)	.176
Previous episodes MDD, median (IQR)	4.0 (3.0)	4.0 (2.25)	4.0 (3.0)	.412
Depressive symptomatology HDRS_17_	3.53 (2.8) (n = 138)	3.50 (2.6) (n = 56)	3.55 (2.9) (n = 82)	.416
Depressive symptomatology (IDS-SR)	17.61 (10.2) (n = 134)	19.90 (10.7) (n = 52)	16.16 (9.6) (n = 82)	.176
Severity last episode				
* Minor (%)*	21/138 (15.2)	5/56 (8.9)	16/82 (19.5)	.214
* Moderate (%)*	76/138 (55.1)	32/56 (57.1)	44/82 (53.7)	
* Severe (%)*	41/138 (29.7)	19/56 (33.9)	22/82 (26.8)	
Daily stress, mean (SD)	(n = 128)	(n = 48)	(n = 80)	
* Dependent frequency*	9.62 (5.0)	9.56 (4.9)	9.65 (5.0)	.881
* Dependent intensity*	12.61 (9.0)	11.65 (8.7)	13.19 (9.2)	.601
* Independent frequency*	6.00 (3.3)	5.58 (3.1)	6.18 (3.4)	..667
* Independent intensity*	7.27(5.2)	6.54 (4.4)	7.70 (5.6)	.137
Childhood adversity no. (%)				
* Loss of a parent*	11/131 (8.4)	4/51 (7.8)	7/80 (8.8)	.855
* Physical abuse*	16/131 (12.2)	4/51 (7.8)	12/80 (15.0)	.223
* Sexual abuse*	19/131(14.5)	8/51 (15.7)	11/80 (13.8)	.759

*Note*, ^a^ p-value based on chi-square statistic for categorical variables and analyses of variance for continuous variables and the Mann-Whitney U for previous episodes of MDD ADM  =  Antidepressant medication; MDD  =  Major Depressive Disorder; HDRS_17_  = 17-item Hamilton Rating Scale for Depression; IDS  =  Inventory of Depressive Symptomatology

Because participants in trial A had to use antidepressants continuously in the last six months to be included in the study, this led to significant differences with regard to ADM use and the rate of visiting a general practitioner between trial A and B. ADM use was not related to any of the daily stress subscales, and depressive symptoms at three month follow-up (all *p*'s>.05). However, ADM use was related to a higher level of baseline depressive symptoms (*p*<.001). We controlled for these baseline differences by including the treatment group (continuation of ADM or TAU), as a variable in the regression analysis.

The data of the continuation of ADM condition of trial A and the TAU condition of trial B were merged. In the combined TAU and continuation of ADM group we estimated the pooled standard deviation (SD) of the IDS-SR_30_ to be 13. Given this SD, it was possible to detect moderate or larger effects (d = 0.53 or over) given 40 subjects with and 100 subjects without a history of childhood adversities, a two-sided alpha of 0.05 and a bèta of 0.8.

### Regression analyses

Depressive symptoms at baseline and three month follow-up, did not significantly differ in participants with,-and without the experience of childhood adversity (Table 2). Childhood adversity did not significantly predict depressive symptoms at three months follow-up (B = 4.073, t = 1.522, *p* = 0.128, 95% CI  = −1.18–9.33). Addition of any of the other variables in the next steps did not lead to any significant changes in this model. The R^2^ and adjusted R^2^ are small (respectively 0.048 and 0.019), indicating that only a small part in the variance of depressive symptoms is explained by the included variables.

**Table 2:** Hierarchical regression model of the TAU and continuation of ADM group for the prediction of depressive symptoms at 3-month follow-up from childhood adversity (n = 138).

Variable		?	t	95% CI	R^2^ Change
Step 1					0.023
	(Constant)	19.535	14.044**	[16.81, 22.26]	
	CA presence	4.073	1.522	[−1.18, 9.33]	
Step 2					0.007
	(Constant)	19.727	4.967**	[11.94, 27.51]	
	CA presence	4.035	1.504	[−1.23, 9.30]	
	Female gender	1.553	0.629	[−3.29, 6.40]	
	Treatment group	−0.874	−0.371	[−5.50, 3.75]	
Step 3					0.002
	(Constant)	15.540	3.154**	[5.88, 25.20]	
	CA presence	4.046	1.515	[−1.20, 9.29]	
	Female gender	1.029	0.417	[−3.81, 5.87]	
	Treatment group	−0.827	−0.353	[−5.42, 3.77]	
	Number of previous episodes	3.176	1.430	[−1.18, 7.53]	

*Note*, CA  =  Childhood adversity; R^2^ final model  = 0.048, Adjusted R^2^ = 0.012.

* p<0.05.

** p <0.01.

The presence of childhood adversity was not related to any of the daily stress subscales(*r* = −.007–.087), and the means and standard deviations of the four daily stress subscales did not significantly differ in participants with-, and without the experience of childhood adversity (all *p*'s >.05) (Table 3) Therefore, we did not proceed to the analysis of a mediating role of daily stress.

**Table 3:** Depressive symptoms at baseline and three-month follow-up in participants with,-and without childhood adversity (n = 138).

Variable	With Childhood Adversity	Without Childhood Adversity	*p* ^a^
Depressive symptomatology (IDS-SR) baseline, mean (SD)	19.82 (10.3)	16.86 (9.8)	.132
Depressive symptomatology (IDS-SR) follow-up, mean (SD)	23.54 (12.4)	19.53 (13.2)	.136
Intensity dependent stress, mean (SD)	13.00 (9.9)	12.47 (8.7)	.233
Intensity independent stress, mean (SD)	7.82 (5.0)	7.07 (5.3)	.956
Frequency dependent stress, mean (SD)	9.42 (4.6)	9.68 (5.1)	.564
Frequency independent stress, mean (SD)	5.94 (3.2)	9.96 (3.3)	.923

*Note*, ^a^p-value based on analyses of variance; IDS-SR * = * Inventory of Depressive Symptomatology.

#### Daily stress as a predictor of depressive symptoms at follow-up

All baseline daily stress subscales showed moderate to high correlations among themselves (*r* = .414–.819, *p*<0.01), and with baseline depressive symptoms (*r* = .308–.399, *p*<0.01). To prevent the loss of explained variance in depressive symptoms, four separate regression analyses were performed for each daily stress subscale (Tables 4–7). Both the reported intensity of independent- and dependent daily stress significantly predicted depressive symptoms at three months follow-up (respectively: B = 0.470, t = 2.046, *p* = 0.041, 95% CI  = 0.02–0.92; B = 0.291, t = 2.202, *p* = 0.028, 95% CI  = 0.03–0.55). After adjustments in the next steps, these results did not change significantly. Frequency of independent and dependent daily stress did not significantly predict depressive symptoms and again these results did not significantly change after adjustments. The R^2^ of all the total models was small (0.044–0.073), as were the adjusted R^2^ of the total models (0.015–0.045), meaning only a small part of the variation in prospectively depressive symptoms in remitted recurrently depressed patients was explained by the variables in the model. Performing the analyses in the original dataset did not lead to any substantial differences in results.

**Table 4:** Hierarchical regression model of the TAU and continuation of ADM group for the prediction of depressive symptoms at 3-month follow-up from independent stress intensity (n = 138).

Variable		?	t	95% CI	R^2^ Change
Step 1					0.039**
	(Constant)	17.244	8.423**	[13.23, 21.26]	
	Independent stress, intensity	0.470	2.046*	[0.02, 0.92]	
Step 2					0.009
	(Constant)	16.185	3.609**	[7.39, 24.98]	
	Independent stress, intensity	0.488	2.086*	[0.03, 0.95]	
	Female gender	2.244	0.910	[−2.59, 7.08]	
	Treatment group	−0.415	−0.177	[−5.01, 4.18]	
Step 3					0.025
	(Constant)	10.768	1.967*	[0.33, 24.50]	
	Independent stress, intensity	0.533	2.279*	[0.07, 0.99]	
	Female gender	1.676	0.684	[−3.13, 6.48]	
	Treatment group	−0.315	−0.136	[−4.87, 4.24]	
	Number of previous episodes	3.785	1.711	[−0.55, 8.12]	

*Note*: R^2^ final model  = 0.073, Adjusted R^2^ = 0.045.

* p <0.05.

** p <0.01.

**Table 5:** Hierarchical regression model of the TAU and continuation of ADM group for the prediction of depressive symptoms at 3-month follow-up from dependent stress intensity (n = 138).

Variable		?	t	95% CI	R^2^ Change
Step 1					0.044
	(Constant)	17.024	8.261**	[12.98, 21.07]	
	Dependent stress, intensity	0.291	2.202*	[0.03, 0.55]	
Step 2					0.005
	(Constant)	17.001	3.936**	[8.53, 25.47]	
	Dependent stress, intensity	0.284	2.138*	[0.02, 0.54]	
	Female gender	1.257	0.514	[−3.54, 6.05]	
	Treatment group	−0.525	−0.223	[−5.13, 4.08]	
Step 3					0.016
	(Constant)	13.218	2.542*	[3.02, 23.42]	
	Dependent stress, intensity	0.276	2.086*	[0.02, 0.54]	
	Female gender	0.781	0.319	[−4.02, 5.58]	
	Treatment group	−0.492	−0.210	[−5.08, 4.09]	
	Number of previous episodes	2.954	1.343	[−1.36, 7.27]	

*Note*: R^2^ final model  = 0.064, Adjusted R^2^ = 0.036.

* p<0.05.

** p<0.01.

**Table 6:** Hierarchical regression model of the TAU and continuation of ADM group for the prediction of depressive symptoms at 3-month follow-up from independent stress frequency (n = 138).

Variable		?	t	95% CI	R^2^ Change
Step 1					0.022
	(Constant)	17.321	6.907**	[12.40, 22.24]	
	Independent stress, frequency	0.561	1.554	[−0.15, 1.27]	
Step 2					0.009
	(Constant)	16.569	3.459**	[7.17, 25.96]	
	Independent stress, frequency	0.591	1.609	[−0.13, 1.31]	
	Female gender	2.138	0.860	[−2.74, 7.01]	
	Treatment group	−0.617	−0.261	[−5.25, 4.01]	
Step 3					0.023
	(Constant)	11.292	1.960	[−0.01, 22.59]	
	Independent stress, frequency	0.659	1.800	[−0.06, 1.38]	
	Female gender	1.598	0.646	[−3.25, 6.45]	
	Treatment group	−0.531	−0.227	[−5.13, 4.06]	
	Number of previous episodes	3.631	1.631	[−0.74, 8.00]	

*Note*: R^2^ final model  = 0.054, Adjusted R^2^ = 0.026.

* p <0.05.

** p <0.01.

**Table 7:** Hierarchical regression model of the TAU and continuation of ADM group for the prediction of depressive symptoms at 3-month follow-up from dependent stress frequency (n = 138).

Variable		?	t	95% CI	R^2^ Change
Step 1					0.018
	(Constant)	17.440	6.703**	[12.34, 22.54]	
	Dependent stress, frequency	0.337	1.427	[−0.13, 0.80]	
Step 2					0.007
	(Constant)	17.543	3.800**	[8.49, 26.60]	
	Dependent stress, frequency	0.339	1.428	[−0.13, 0.80]	
	Female gender	1.673	0.675	[−3.19, 6.53]	
	Treatment group	−0.888	−0.376	[−5.52, 3.74]	
Step 3					0.018
	(Constant)	13.361	2.430*	[2.58, 24.15]	
	Dependent stress, frequency	0.339	1.435	[−0.12, 0.80]	
	Female gender	1.150	0.465	[−3.70, 6.00]	
	Treatment group	−0.841	−0.358	[−5.45, 3.76]	
	Number of previous episodes	3.171	1.428	[−1.18, 7.53]	

*Note*: R^2^ final model  = 0.044, Adjusted R^2^ = 0.015.

* p <0.05.

** p <0.01.

## Discussion

Our first research aim was to investigate the influence of childhood adversity on the depressive symptoms three months after remission and whether adult daily stress was a mediator of this potential influence. We found no support for our hypothesis that childhood adversity predicts depressive symptoms. Additionally, in contrast to our hypothesis, childhood adversity was not related to daily stress in later life either. This is at odds with previous research where childhood adversity led to higher later life stress, which in turn predicted the onset of depression [31], [32]. However, this is the first investigation of the effect of childhood adversity on daily stress and prospectively measured depressive symptoms in recurrently depressed patients while remitted. Importantly, these findings have to be interpreted in the context of our highly recurrent study sample. With a median of four previous depressive episodes (IQR  = 3.0), a potential effect of childhood adversity on risk of relapse might have been overshadowed. Unfortunately, we cannot compare this to other related studies, because in comparable studies overall the information on the mean or median number of previous depressive episodes was not mentioned [51]–[54].

Alternatively, different pathways could lead from childhood adversity to depressive relapse. Cognitive variables, such as dysfunctional attitudes, may play a role given that they develop early in life and the occurrence of childhood adversities is presumed to negatively influence their development [55], [56]. Evidence for this pathway was found in previous research in patients with a history of depression, where a negative cognitive style was a mediator in the relation between childhood adversity and stress generation [34]. More research is needed to specify this potential pathway in recurrent depression.

A final research aim was to examine if daily stress was predictive of depressive symptoms measured three months after baseline remission. In line with previous research [31], we found intensity of dependent and independent daily stress to be predictive of subsequent depressive symptoms after remission. Although we expected only dependent stress to be predictive, our finding is consistent with the study of Monroe et al. [30]. In their study, 126 recurrently depressed patients were followed over three years of maintenance treatment, where ‘subject focused independent daily stress’ (an event that happens to a person self instead of an acquaintance or relative), was predictive of depressive relapse. Abramson, Seligman & Teasdale [57], suggested that independent stress poses a risk for depressive relapse because of the lack of control on individual experiences when independent events occur which heightens the stress response.

### Strengths and Limitations

While it is an advantage to study this high risk of relapse patient group to determine whether childhood adversity and daily stress influence the return of depression, the downside is that the experience of previous episodes could make the detection of pathways from childhood adversity to depressive relapse difficult. The following limitations have to be taken into account. First, the level of depressive symptoms at three-month follow-up is relatively low since patients were remitted at study start which could make finding an effect difficult. Second, the limited follow-up time of three months and relatively small sample size may lower the chance of detecting an effect. However, based on the number of patients with-, versus without exposure to childhood adversity, detection of a moderate to large effect seemed possible. Although this standard deviation was derived out of our own study, it was comparable to other MDD populations [58]. Third, while retrospective assessment of childhood adversity is representative, irrespective of presence of mental illness even up to 20 years [59], self-report is said to lead to less strong associations than contextual measures [60]. Alloy, Liu and Bender [61], state that using a self-report checklist as assessment tool of stressful life events could lead to more interpretative biases by patients and the lack of contextual information makes it hard to differentiate between dependent and independent events. Furthermore, information about the intensity and frequency of childhood adversity is missing, but could be decisive with regard to long-lasting influences on stress and depression. Fourth, there is a wide variety of other types of childhood adversity that were not assessed but could be of importance. For example emotional neglect and emotional abuse were not assessed in our study, although they are potentially important to the prognosis of depression [10], [12], [13]. Additionally previous research in patients with a history of depression showed that specifically emotional neglect was the significant predictor of prospective negative dependent events [34]. Fifth, we used four separate regression analyses to examine the associations between the four daily stress subscales and depressive symptoms. However, these four hypotheses may not be completely independent. After a Bonferroni correction which is considered conservative, the intensity of dependent-, and independent daily stress would not be significant predictors of depressive symptoms at follow-up anymore. Bearing in mind that the use of adjustment procedures for multiple comparisons has been criticized for, amongst others, falsely reporting no significant associations [62], no adjustments are needed for multiple comparisons. Nevertheless, we stress the need for cautious interpretation of the results. Sixth, the participants in this study were recruited for two studies that differed with respect to ADM use and the mean severity of the previous depressive episode. Although we controlled for treatment group, these differences still may have influenced the results. Seventh, the low level of variance in depressive symptoms explained by intensity of daily stress suggests the existence of other predictors of depressive symptoms. Finally, it cannot be ruled out that the observed relationship between daily stress and depressive symptoms at follow-up is actually reverse, i.e. baseline depressive symptoms cause baseline daily stress and depressive symptoms at follow-up. However, in this study we were interested in the prediction of depressive symptoms from daily stress and we leave the possibility that the relationship is partially explained by baseline depressive symptoms as an intermediate variable open.

### Conclusion and future directions

Our study suggests that the perceived intensity of daily stress is a predictor of depressive symptoms three months after remission, which heightens the risk of relapse in recurrently depressed patients. The intensity of daily stress is potentially modifiable and could therefore be a treatment aim in the prevention of depressive relapse, irrespective of experienced childhood adversity. There is an indication that PCT reduces the negative influence of daily stress on depressive relapse [22], [23], but more information on the course of daily stress is required to examine long-term treatment effects on daily stress and relapse. Other studies, with a larger sample size and a longer follow-up time, are needed to replicate these findings and to examine other potential pathways of childhood adversity to depressive relapse, including cognitive variables.

## Supporting Information

Checklist S1 **CONSORT checklist.**
(DOC)

Protocol S1 **Trial protocol (trial B).**
(PDF)

Protocol S2 **Trial protocol (trial A).**
(PDF)
