# Supplementary material for: The Scars of Childhood Adversity: Minor Stress Sensitivity and Depressive Symptoms in Remitted Recurrently Depressed Adult Patients
Source: PLoS One. 2014 Nov 13;9(11):e111711. doi: 10.1371/journal.pone.0111711 (PMC4230942; doi:10.1371/journal.pone.0111711)
Supplement: Protocol S2 — Trial protocol (trial A). (PDF) [file pone.0111711.s003.pdf]

Bockting et al. BMC Psychiatry 2011, 11:8
http://www.biomedcentral.com/1471-244X/11/8

BMC Psychiatry

STUDY PROTOCOL Open Access

Disrupting the rhythm of depression: design and protocol of a randomized controlled trial on preventing relapse using brief cognitive therapy with or without antidepressants

Claudi LH Bockting[1*], Hermien J Elgersma[1,2], Gerard D van Rijsbergen[1], Peter de Jonge[3], Johan Ormel[3], Erik Buskens[4], A Dennis Stant[4], Peter J de Jong[1], Frenk PML Peeters[5], Marcus JH Huibers[6], Arnoud Arntz[6], Peter Muris[7], Willem A Nolen[3], Aart H Schene[8], Steven D Hollon[9]

Abstract

Background: Maintenance treatment with antidepressants is the leading strategy to prevent relapse and recurrence in patients with recurrent major depressive disorder (MDD) who have responded to acute treatment with antidepressants (AD). However, in clinical practice most patients (up to 70-80%) are not willing to take this medication after remission or take too low dosages. Moreover, as patients need to take medication for several years, it may not be the most cost-effective strategy. The best established effective and available alternative is brief cognitive therapy (CT). However, it is unclear whether brief CT while tapering antidepressants (AD) is an effective alternative for long term use of AD in recurrent depression. In addition, it is unclear whether the combination of AD to brief CT is beneficial.

Methods/design: Therefore, we will compare the effectiveness and cost-effectiveness of brief CT while tapering AD to maintenance AD and the combination of CT with maintenance AD. In addition, we examine whether the prophylactic effect of CT was due to CT tackling illness related risk factors for recurrence such as residual symptoms or to its efficacy to modify presumed vulnerability factors of recurrence (e.g. rigid explicit and/or implicit dysfunctional attitudes). This is a multicenter RCT comparing the above treatment scenarios. Remitted patients on AD with at least two previous depressive episodes in the past five years (n = 276) will be recruited. The primary outcome is time related proportion of depression relapse/recurrence during minimal 15 months using DSM-IV-R criteria as assessed by the Structural Clinical Interview for Depression. Secondary outcome: economic evaluation (using a societal perspective) and number, duration and severity of relapses/recurrences.

Discussion: This will be the first trial to investigate whether CT is effective in preventing relapse to depression in recurrent depression while tapering antidepressant treatment compared to antidepressant treatment alone and the combination of both. In addition, we explore explicit and implicit mediators of CT.

Trial registration: Netherlands Trial Register (NTR): NTR1907

Background

Major depressive disorder (MDD) is projected to rank second on a list of 15 major diseases in terms of burden of disease in 2030 [1]. The major contribution of MDD to disability and health care costs is largely due to its highly recurrent nature [2,3]. Accordingly, efforts to reduce the disabling effects of depression should shift to preventing recurrences, especially in patients at high risk of recurrence. Several international guidelines (e.g., [4,5]) report that patients remitted from prior depressive episodes belong to such high risk groups. The preventive strategy globally suggested, i.e. continuation of antidepressants (AD) for years, is certainly not in line with

* Correspondence: c.l.h.bockting@rug.nl
[1]Department of Clinical Psychology, Groningen University, The Netherlands
Full list of author information is available at the end of the article

BioMed Central
© 2011 Bockting et al; licensee BioMed Central Ltd. This is an Open Access article distributed under the terms of the Creative Commons Attribution License (http://creativecommons.org/licenses/by/2.0), which permits unrestricted use, distribution, and reproduction in any medium, provided the original work is properly cited.

the fact that 70-80% of the patients are not willing to take AD for a long period of time [6,7]. Many patients prefer psychological interventions instead. Moreover, continuation of AD has clear limitations. First, non-adherence in AD-users is common (50%, [5-8]). It may also be contraindicated because of somatic illness or side effects. Further, patients' protection from recurrence ceases on discontinuation of AD [9]. Moreover, a recent meta-analysis indicates that with increasing number of previous episodes, patients develop resistance against the prophylactic properties of AD [10]. Finally, the optimal duration of the maintenance phase has not been studied adequately since few studies reported follow-up periods longer than 1 year.

Meta-analyses indicate that cognitive therapy (CT) is not only an effective treatment of MDD, but can also be used as an effective preventive intervention [10-17]. Sequential treatment in which CT is started after remission has proven to be effective in preventing recurrences in patients with recurrent MDD (e.g. for a meta-analysis see [12]). In a previous multicenter RCT enrolling remitted recurrently depressed patients, the efficacy and cost-effectiveness of CT was evaluated added to treatment as usual (TAU) compared with TAU alone [18]. In line with other studies on this preventive CT, CT was effective [6] and cost-effective [Bockting CLH, Dijkgraaf MGW, Hakaart-van Roijen L et al. Cost-effectiveness of relapse-prevention cognitive therapy in recurrent depression: a two year study. Submitted] in preventing recurrences over a 2-year follow-up and also over 5.5 years in patients with multiple previous episodes [19]. In this case TAU included several types of aftercare; e.g. continuation of AD, non-controlled tapering and discontinuation of AD, both in primary and secondary care. Recently, the preventive effects of CT in primary care have been confirmed in patients who have experienced multiple previous episodes [20]. Since in our prior studies we did not plan a controlled tapering of AD treatment versus AD continuation or the combination with CT, we could not examine whether CT while tapering AD or combining it with AD is an effective strategy in preventing relapse in recurrent depression [5,21]. A previous study [22] randomly assigning remitted recurrently depressed patients (n = 40) to CT or clinical management, while withdrawing AD, reported a 50% reduction in relapse rate in CT vs. clinical management over 6-year follow-up (40% vs. 90%). This study suggests long term effects of CT in patients that stopped AD. There is convincing evidence, based on a recent meta-analysis [12], for the preventive effect of CT, and some evidence for specific forms of CT (like Mindfulness Based Cognitive Therapy; MBCT). In the UK a recent study (n = 123) compared CT (MBCT) while tapering AD, against AD in recurrent depression and revealed relapse rates over 1 year of 47% for CT while 60% of the patients relapse in the group that used AD [23].

Trial objectives and Purpose

The objective of this study is to examine whether CT is an effective preventive strategy in reducing relapse/recurrence while tapering AD compared to continuation of AD versus the combination of maintenance AD with CT.

Alongside this effect study an economic evaluation will be performed. In addition, the study explores potential moderators to examine what works for whom. Potential mediators will be examined to explore the working mechanism of preventive CT by assessing implicit and explicit beliefs, coping related factors, symptoms, stressful life events, and their association to risk of relapse to depression, before, between and after treatment and during the follow-up period.

Methods/Design

In this multi-center randomized controlled three-arm trial with a sample size of 276 participants an 8 session group CT with guided tapering of AD, will be compared to continuation of maintenance AD use versus the combination of CT with maintenance AD in remitted patients with recurrent MDD. In doing so we stratify on the number of previous episodes and type of aftercare. The effectiveness and cost-effectiveness (societal perspective) of the interventions will be examined with a follow-up of minimal 15 months.

We undertake randomization by telephone to the Psychiatry department of the University Medical Center of Groningen (UMCG). The number of previous depressive episodes and type of care are delineated for stratification reasons.

We monitor the primary outcome (relapse) over a period of minimal 15 months. Assessments by trained assessors who are blind to treatment allocation (and whose blindness is checked within each assessment session) take place directly after the start of the treatment, at three months, nine and 15 months. In between there is additionally a self report assessment at 1.5 months for the research question on mediation. For an overview of the study's procedure see Figure 1.

The interventions

CT-arms

Patients in the two arms including CT (with tapering of AD and with continuation of AD) receive the proposed intervention (8 weekly group sessions). CT helps patients to identify and change presumed vulnerability factors of recurrence, i.e. dysfunctional beliefs, in line with traditional CT in the acute phase of depression.

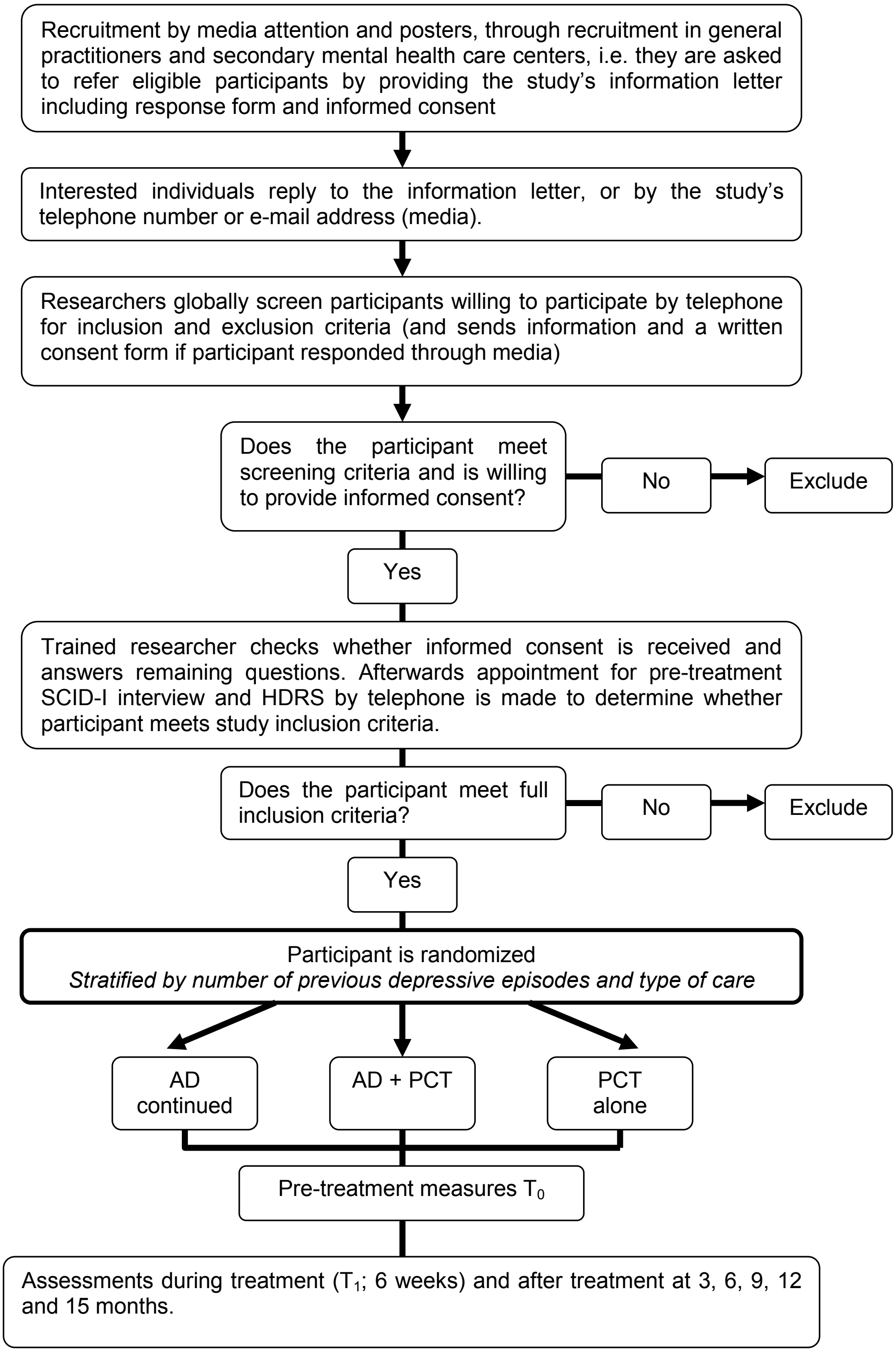

Figure 1 ***Flow-chart of the study***. AD = antidepressant, PCT = preventive cognitive therapy.

Traditional CT assumes that change of the negative content of thoughts and attitudes are crucial features of successful interventions. More recent cognitive theories of depression, however, emphasize the role of information-processing biases and suggest that these biases are at the core of depression vulnerability and enhanced stress reactivity, via enhancing the elaboration of negative material in memory and reasoning (e.g. [24]). If indeed a difficulty to disengage from negative material is critically involved in the return of complaints, it would

be important to see whether CT is not only successful in changing content of thoughts and memory associations, but also helps to increase inhibitory control, thereby disrupting the processes of negative emotions and ruminative negative thinking patterns [25].

AD-arms

In the treatment arms where AD will be continued, GP's and psychiatrists will be advised to continue AD prescription at minimal required adequate used dosage (≥ 20 mg Fluoxetine equivalent; [6] as recommended by national guidelines [26]. In the treatment arm including guided tapering of AD GP's and psychiatrists will be advised to taper AD in 4 weeks to prevent withdrawal symptoms. In this arm patients will be asked for an intention to taper AD. The patient is allowed to start AD again at any time during the study (this will be monitored).

We will assess compliance and adherence to AD use with the Medication Adherence Scale [27]. We also assess adherence to CT prescription of homework and presence at the sessions. Patients will be encouraged to use medication/do homework as prescribed and doctors/psychiatrists will be encouraged to prescribe therapeutic dosages, as well as discuss problems with adherence frequently.

Sample size

In total 276 patients will be recruited. This sample size provides 80% power (alpha 0.05, two sided) to demonstrate in survival analysis a clinical significant difference of 20% in relapse/recurrence between AD versus AD+CT (relapse 50% in AD patients versus 30% in AD+CT) patients after 15 months, based on a recent meta-analysis on AD versus placebo [10]. Taking into account 15% attrition, for this comparison 2 × 98 participants will be needed. To demonstrate a clinically significant difference of 15% in relapse/recurrence rate (relapse rate AD: 50%) between the two treatment arms with continuation of AD versus the CT arm while tapering continuation AD additional 80 participants will be needed.

Inclusion criteria

We will include patients:

-with at least two previous depressive episodes in the past five years.

-who are currently in remission according to DSM-IV criteria, for longer than 8 weeks and no longer than 2 years.

-that have a current score of <10 in the 17 item Hamilton Rating Scale for Depression (HRSD; in line with other prevention studies, e.g. [12,18,28]

-for whom the last episode is at least 2 months and no longer than 2 years ago.

-that have been remitted on antidepressant treatment and use AD at entry in the study (delivered in primary or secondary care) for at least 6 months.

Exclusion criteria

Exclusion criteria are: current mania or hypomania or a history of bipolar illness, any psychotic disorder (current and previous), organic brain damage, alcohol or drug dependency/abuse, predominant anxiety disorder.

Eligibility, informed consent and baseline assessments

The target population is a high-risk group as identified in several guidelines, (e.g., [4,5]) that consumes a considerable amount of health care and for whom initial benefits of AD may be lost in the long run. Relapse rates rise with increasing numbers of previous episodes up to 70% in 5 years [28]. In our previous study, we observed up to 62% recurrences within 2 years [29].

A highly similar recruitment procedure will be used as in our previous study in which we recruited via media and via referrals from general practitioners or medical specialists in secondary mental health care [18,19]. The study will be conducted by a team of clinical psychologists of the department of Psychology at the University of Groningen in collaboration with psychologists of the Rotterdam University and Maastricht University and psychiatrists of the departments of Psychiatry of the University of Amsterdam (AMC) and the Groningen University (UMCG). We collaborate with 16 mental health care sites in The Netherlands. The group intervention will be performed in several cities. Therapists of the sites will be trained with a CT manual to promote treatment integrity.

Informed consent

We inform patients about the study before they come into the study in two ways. First, by informing the patient through their therapist: if a therapist wants to inform the patient themselves, the patient then receives the information via the therapist and is given a letter containing all the information. If the patient is interested in participating then the participant contacts the researcher. Subsequently, the researcher checks whether the participant understands all aspects of the trial. If they agree to enter the trial, they complete a copy of the consent form and send it back to the researcher.

The second procedure we use is by directly informing the patient in case the patient contacts the researcher (mostly informed by media or by their former therapist/GP with a letter, by advertisements or interviews). Subsequently, the researcher informs the patient, he/she receives the information in a letter with all the information in it. If the patient is still interested in participating

after reading the information then the participant contacts the researcher. The researcher checks whether the patient understands all aspects of the trial. If the patient agrees to enter the trial, he/she completes a copy of the consent form and sends it to the researcher.

We remind participants that they can withdraw from the trial at any time and that this will have no consequences for their treatment as usual.

We ask consenting participants to provide information about their socio-demographic background and assess their eligibility in more detail using semi-structured clinical interviews (SCID-I) and self-completed questionnaires. The researchers assess current and past diagnostic status using the Structured Clinical Interview for DSM-IV (SCID, [30]) and the Hamilton Rating Scale for Depression (HRSD, [31]). They ask participants to describe past and current treatments for depression, their attribution of relapse and recurrence and use of AD. If participants meet all inclusion and none of the exclusion criteria for the study, they enter the study.

For the baseline assessment, we ask participants themselves to complete the web based self report questionnaires in two packages: explicit and implicit measures. The first part with assessments starting directly within a week for the AD continuation only arm, and for the other arms briefly before starting preventive CT. The second part of the assessment include implicit measures that will be offered within 2 days after completion of the self report assessments measures. Used self report measures are: the Inventory of Depressive Symptomatology, IDS-SR [32], negative life events (Life events questionnaire, LGV [33], self-esteem (Rosenberg's Self-esteem Scale [34]), personality pathology (Personality Diagnostic Questionnaire, PDQ-4 [35]), somatic complaints (LKV [36]), everyday problems (EPCL [37]), hypomania (HCL-32 [38]), direct and indirect costs (TIC-P [39]) and Medication Adherence Questionnaire (MAQ, [27]). a measure of general quality of life (Euro-QOL EQ-5 D [40]), rumination (Ruminative Responses Subscale of the Response Styles Questionnaire, RSQ [41]), dysfunctional attitudes (Dysfunctional Attitudes Scale, DAS [42]) and cognitive reactivity using the LEIDS [43]), acceptance (Acceptance and Action Questionnaire, AAQ [44]) and coping (Utrecht Coping List, UCL [45]). After 6 weeks this set will be repeated with the exception of the TIC-P, LGV, PDQ, MAS and EQ-5 D. During follow-up every three months the following self report assessments will be repeated: IDS-SR, HCL-30, TIC-P, EPCL, and EQ-5 D. For a complete overview of the assessments see table 1.

Outcome measures

Primary outcome effectiveness: time related proportion of depression relapse/recurrence in a survival analysis (Cox regression) over a follow-up period of minimal 15 months using DSM-IV-TR criteria as assessed by the Structured Clinical Interview for DSM-IV (SCID, telephonic version, [46]) at 3 months, 9 months and 15 months (current depressive symptomatology and previous 3 and 6 months).

For potential differential (illness related, stress-related and cognitive-related) predictors and mediators the following self report measures will be used at baseline, at 1 and at 3 months (internet based): Inventory of Depressive Symptomatology (IDS-SR; [32]), Negative Life Events Questionnaire [33], Everyday Problem Checklist (EPCL; [37]), Ruminative Responses Subscale of the Response Styles Questionnaire (RSQ; [41]), Dysfunctional Attitude Scale, (DAS-A; [42]), LEIDS [43] and to assess nonadherence to AD with the Medication Adherence Questionnaire (MAQ; [27]). To enable calculating quality adjusted life years required for the economic evaluation, also the EQ5 D also will be administered every 3 months [40]. To test whether CT and/or AD affect implicit attitudes and attentional bias differentially and whether residual difficulty to disengage and residual dysfunctional implicit attitudes are related to the return of depressive symptoms an Implicit Association Test (IAT; [47]) that is also used in the Netherlands Study of Anxiety and Depression http://www.nesda.nl will be used to assess implicit attitudes. A rapid serial visual presentation (RSVP; [48]) task will be used to assess the difficulty to disengage from negative information. Difficulty to disengage will be indexed by the magnitude of the attentional blink when negative self descriptors are presented as the first target and neutral words as the second.

For an overview of the assessments at baseline, in between- and post treatment and follow up assessments see table 1.

Withdrawal

Participants can withdraw from treatment or from the study at any time. Nevertheless we ask those who withdraw from the trial treatment (CT or CT+AD or AD alone) if they are willing to attend all the remaining research appointments or at least to provide minimal data.

Safety monitoring and reporting

The trial protocol has been approved by an independent medical ethics committee for all included sites (METIGG). Eligible people will be included as participants in the trial only after informed consent has been obtained.

We record and report suspected serious adverse events to the Multi-centre Ethic Committee (METIGG) according to their individual guidelines.

Table 1 Overview of assessments

Measure	Description	T_0	T_1	T_2	T_3	T_4	T_5	T_6*
IDS-SR	Depressive symptoms	+	+	+	+	+	+	+
RSQ	Ruminative responses	+	+	+				+
EQ-5D	Quality of life	+		+	+	+	+	+
DAS	Dysfunctional Attitudes	+	+	+				+
AAQ	Experiential acceptance and avoidance	+	+	+				+
UCL	Coping	+	+	+				+
LGV	Life-events	+		+				+
Self-esteem	Self-esteem	+	+	+				+
PDQ-4+	Personality	+						
LKV	Somatic complaints	+						+
EPCL	Everyday problem list	+	+	+	+	+	+	+
HCL-32	Hypomania	+	+	+	+	+	+	+
TIC-P	Direct/indirect costs	+		+	+	+	+	+
LEIDS	Dysfunctional attitudes	+	+	+				+
SCID-I	DSM-IV-TR Axis I disorders	+		+		+		+
HDRS	Depressive symptoms and severity	+		+		+		+
IAT	Implicit associations	+		+				
RSVP	Ability to disengage from negative information	+		+				

T0 = Baseline, T1 = ± 1,5 month, T2 = 3 months, T3 = 6 months, T4 = 9 months, T5 = 12 months, T6 = 15 months.

Analysis

Cox regression analysis (survival analysis) will be performed, including as covariates the stratification variables that will be used in randomization, and 2 additional variables, i.e.: number of previous episodes, type of care (primary/secondary). Analysis will be conducted by intention to treat, including all subjects randomized in the study (including dropouts and patients who did not taper AD), and per protocol, including only subjects satisfying protocol treatment (up to the point in the study where they failed to do so). Statistical significance will be set at $P < .05$. Mixed-model analysis will be used for the other variables, including baseline values of the dependent variable as a covariate in all analyses. We shall use stress measures and implicit and explicit cognitive measures to explore the extent to which they mediate relapse and recurrence during treatment and follow up.

For the economic evaluation the balance between costs and health outcomes will be compared across strategies using a societal perspective. Primary outcome measure: the number of depression-free days. Both short-term and long-term consequences will be compared. Additionally, Quality Adjusted Life Years will be used as outcome.

Discussion

Recurrent depression is highly prevalent and reducing relapse and recurrence is therefore essential. This trial will be the first comparison of CT while tapering AD versus continuation of AD versus the combination of both. Apart from the evaluation of the effectiveness and cost-effectiveness, we examine what works for whom. The most frequently used preventive strategy, i.e. continuation of AD for years, is not in line with what patients do and want (70-80% of the patients are not willing to take AD long enough; [6,7]). Many patients prefer psychological interventions instead. The results of this study might improve better patient by treatment matching by examining the effects of divers preventive strategies and explore what strategy is best for a person with specific characteristics. In addition, mediation variables will be examined to get more insight into the essential ingredients of the preventive CT used. This might lead the development of more targeted interventions.

In summary, MDD has a highly recurrent nature [2]; accordingly, efforts to reduce the disabling effects of depression should shift to preventing recurrence, especially in patients at high risk of recurrence. The development and evaluation of alternative preventive strategies and their specific working mechanism, apart from AD, are needed to at least disrupt the rhythm of depression for this large patient group. This trial will contribute to improved and more efficient therapeutic regimens to prevent relapse and recurrence in depression.

Appendix 1: Statistical Analysis Plan

The primary outcome measure will be the time to relapse or recurrence meeting DSM-IV criteria for a major depressive episode (American Psychiatric Association, 1994) on the Structured Clinical Interview for

DSM-IV (SCID, [46]). Occurrence of relapse or recurrence (current or since the last assessment point) will be assessed after treatment at 3 months, and at nine months and 15 months thereafter by trained psychologists who are blind to treatment condition. The analysis will be by 'intention to treat' (ITT). The time (in weeks) of relapse or recurrence to Major Depression, as defined above, will be the dependent variable in survival analysis. The treatment group and stratification variables will be used as predictors.

For participants who are lost from the trial, available measures will be used and then censored at the time of their last observation. Since only a participant's first relapse or recurrence to Major Depression will contribute to the survival analysis, the subsequent loss of that participant will not affect the analysis. Participants who miss one or more follow-up assessments, but are then assessed at a later time point will be asked about their current and past symptoms according to SCID diagnostic criteria since their last successful assessment. This will enable us to assess the time to relapse and thus to censoring.

In addition, we use Hamilton Rating Scale for Depression interview (HRSD), to assess the severity of depression at all time points. The other quantitative measures used at baseline, before treatment, and every three months are the IDS-Q, HSRS-E, LKV, PDQ-4, DAS, LEIDS, UCL, RRS, AAQ-II, Self-esteem questionnaire, LGV, EPCL, TIC-P and the EQ5-D (used to compute QALY's). We shall calculate the 'area under the curve' (AUC) of each measure to give a single score.

Mixed-model analysis of covariance (ANCOVA) will be used for the quantitative measures. As covariates we shall use the stratification variables (number of episodes and type of aftercare and treatment group, together with treatment adherence either to continuation AD or tapering AD or to the number of sessions attended). Potential moderators to be examined include gender, number of previous episodes, residual symptoms of depression (e.g. HRSD score and IDS score at baseline) and duration of remission, duration of last episode, familial psychiatric burden, life events (childhood/adult), coping and age of onset.

For mediation analysis, regression will be used to examine pre-post change on CT versus AD alone (binary for the dichotomous outcome of relapse and linear regression for the HRSD score, the DAS, LEIDS and daily hassles score (EDPL) during follow-up and the association of this potential change to outcome (relapse/recurrence) will be explored.

Cost-effectiveness will be evaluated from a societal perspective; costs in and outside the healthcare sector will be part of the analyses. Both short-term and long-term consequences of the studied interventions will be taken into account. For the short term analysis, a time horizon of 15 months will be applied, during which information on costs and health outcomes will be prospectively collected using the TIC-P. The primary outcome measure of the cost-effectiveness analysis is the number of depression-free days as assessed by the SCID. In the additionally planned cost-utility analysis, QALY's (Quality Adjusted Life Years) will be used as the primary outcome measure as assessed by the EQ5-D. Medical costs that will be assessed include costs related to CT, medication use, hospital admissions, and contacts with healthcare professionals. Outside the healthcare sector, various costs of informal care and productivity losses will be included. Unit prices will largely be based on Dutch standard prices [49]. Costs and health outcomes will be discounted in accordance with Dutch guidelines. The bootstrap method will be applied to estimate non-parametric confidence intervals for mean differences in costs between groups. Furthermore, cost-effectiveness acceptability curves will be used to inform decision-makers on the probability that the studied intervention is cost-effective. The long-term consequences of CT versus AD will be addressed by a decision type model, focusing on a period of 20 years. Main aspects of the model will be based on data collected in the prospective part of the trial, literature on the patient population under study, and expert opinions. Important cost types that are expected to demonstrate differences between the two treatment arms over a time frame of 20 years include costs related to AD use, contacts with healthcare professionals, and lost productivity. Also, scenario analyses reflecting the duration of effect will be conducted to evaluate the period of sustained effect which results in a break even between costs and long term savings. The primary health outcome applied in the long-term model will be the QALY. Since QALY's cannot directly be assessed during the 20 years time horizon of the long-term analysis, previously published data on long-term QALY outcomes in comparable patient populations will be used when available (or assumptions will have to be made). Sensitivity analyses will focus on variations of applied probabilities, QALY estimates, and influential cost components.

Acknowledgements
The research is funded by ZONMW: The Netherlands association for Health research and Development (171002401) to CLH Bockting Associate professor of Clinical Psychology and by ZONMW, The Netherlands association for Health research and Development, OOG Grant (100002035) to HJ Elgersma and CLH Bockting. The Netherlands Organization for Scientific Research (NWO) funded this manuscript.

Author details
[1]Department of Clinical Psychology, Groningen University, The Netherlands. [2]Mental health care center Accare, The Netherlands. [3]Department of Psychiatry, University Medical Center Groningen, Groningen University, The

Netherlands. [4]Medical Technology Assessment, Department of Epidemiology, University Medical Center Groningen, Groningen University, The Netherlands. [5]Department of Psychiatry and Neuropsychology, Maastricht University Medical Center, The Netherlands. [6]Department of Clinical Psychological Science, University of Maastricht, The Netherlands. [7]Department of Clinical and Health Psychology, Erasmus University, Rotterdam, The Netherlands. [8]Department of Psychiatry, Academic Medical Center, University of Amsterdam, Amsterdam, The Netherlands. [9]Vanderbilt University, Department of Psychology, Nashville, Tennessee, USA.

Authors' contributions

CB drafted this paper (which was added to and modified by all other authors) and wrote the treatment manual for the used intervention, WN wrote the protocol for tapering AD, all authors (except GvR) contributed to the design of the study and CB and PdJe to the analytic strategy. All authors read and approved the final manuscript.

Competing interests

CB participated in a discussion on treatment for depression for a web-based course of Wyeth once on 1/11/2007. FP received speakers' fees from GlaxoSmithKline, Wyeth, Astra Zeneca, Lundbeck, Eli Lilly, Servier, and Janssen-Cilag. WN received grants from Netherlands Organisation for Health Research and Development, Stanley Medical Research Institute, Astra Zeneca, Eli Lilly, GlaxoSmithKline and Wyeth, he received speaker's fees from Astra Zeneca, Eli Lilly, Pfizer, Servier, Wyeth and was in the advisory board of Astra Zeneca, Cyberonics, Eli Lilly, GlaxoSmithKline, Pfizer and Servier. All other authors declare that they have no competing interests.

Received: 10 December 2010 Accepted: 12 January 2011
Published: 12 January 2011

References

1. Mathers CD, Loncar D: **Projections of global mortality and burden of disease from 2002 to 2030.** *PLoS Med* 2006, **3**:442.
2. Murray CJ, Lopez AD: **Regional patterns of disability-free life expectancy and disability-adjusted life expectancy: global Burden of Disease Study.** *Lancet* 1997, **10(349)**:1347-52.
3. Ormel J, Petukhova M, Chatterji S, Aguilar-Gaxiola S, Alonso J, Angermeyer MC, Bromet EJ, Burger H, Demyttenaere K, de Girolamo G, Haro JM, Hwang I, Karam E, Kawakami N, Lépine JP, Medina-Mora ME, Posada-Villa J, Sampson N, Scott K, Ustün TB, Von Korff M, Williams DR, Zhang M, Kessler RC: **Disability and treatment of specific mental and physical disorders across the world: Results from the WHO World Mental Health Surveys.** *Br J Psychiatry* 2008, **192**:368-375.
4. American Psychiatric Association: *Practice guidelines for the treatment of psychiatric disorders: Compendium 2008* Washington, D.C; 2008.
5. Clinical NICE Depression: *Management of depression in primary and secondary care* The National Collaborating Centre for Mental Health; 2009.
6. Bockting CLH, ten Doesschate MC, Spijker J, Spinhoven Ph, Schene AH, the DELTA study group: **Continuation and maintenance use of antidepressants in recurrent depression.** *Psychoth Psychosom* 2008, **77**:17-26.
7. Meijer WE, Heerdink ER, Leufkens HG, Herings RM, Egberts AC, Nolen WA: **Incidence and determinants of long-term use of antidepressants.** *European J Clin Pharmacology* 2004, **60**:57-61.
8. ten Doesschate MC, Bockting CLH, Schene AH: **Adherence to continuation and maintenance antidepressant use in recurrent depression.** *J Clin Psychiatry* 2009, **70**:63-9.
9. Viguera AC, Baldessarini RJ, Friedberg J: **Discontinuing antidepressant treatment in major depression.** *Harv Rev Psychiatry* 1998, **5**:293-306.
10. Kaymaz N, van Os J, Loonen AJ, Nolen WA: **Evidence that patients with single versus recurrent depressive episodes are differentially sensitive to treatment discontinuation: a meta-analysis of placebo-controlled randomized trials.** *J Clin Psychiatry* 2008, **69**:1423-36.
11. Beck AT: **The current state of cognitive therapy: a 40-year retrospective.** *Arch Gen Psychiatry* 2005, **62**:953-9.
12. Vittengl JR, Clark LA, Dunn TW, Jarrett RB: **Reducing relapse and recurrence in unipolar depression: a comparative meta-analysis of cognitive-behavioral therapy's effects.** *J Consult Clin Psychol* 2007, **75**:475-88.
13. DeRubeis RJ, Hollon SD, Amsterdam JD, Shelton RC, Young PR, Salomon RM, O'Reardon JP, Lovett ML, Gladis MM, Brown LL, Gallop R: **Cognitive Therapy versus Medications in the Treatment of Moderate to Severe Depression.** *Arch Gen Psychiatry* 2005, **62**:409-16.
14. Hollon SD, DeRubeis RJ, Shelton RC, Amsterdam JD, Salomon RM, O'Reardon JP, Lovett ML, Young PR, Haman KL, Freeman BB, Gallop R: **Prevention of relapse following cognitive therapy vs medications in moderate to severe depression.** *Arch Gen Psychiatry* 2005, **62**:417-27.
15. Hollon SD, Stewart MO, Strunk D: **Enduring effects for cognitive behavior therapy in the treatment of depression and anxiety.** *Annu Rev Psychol* 2006, **57**:285-315.
16. Clarke GN, Hornbrook M, Lynch F, Polen M, Gale J, Beardslee W, O'Connor E, Seeley J: **A randomized trial of a group cognitive intervention for preventing depression in adolescent offspring of depressed parents.** *Archives Gen Psychiatry* 2001, **58**:1127-34.
17. Dobson KS, Hollon SD, Dimidijan S, Schmaling KB, Kohlenberg RJ, Gallop R, Rizvi SL, Gollan JK, Dunner DL, Jacobson NS: **Randomized trial of behavioral activation, cognitive therapy, and Antidepressant medication in prevention of relapse and recurrence in major depression.** *J Cons Clin Psychol* 2008, **76**:468-77.
18. Bockting CLH, Schene AH, Spinhoven Ph, Koeter MWJ, Wouters LF, Huyser J, Kamphuis JH: **Preventing recurrence in recurrent depression using cognitive therapy.** *J Cons Clin Psychol* 2005, **73**:647-657.
19. Bockting CLH, Spinhoven Ph, Koeter MWJ, Schene AH: **Long term effects of preventive cognitive therapy in recurrent depression using: 5.5 years follow-up.** *J Clin Psychiatry* 2009, **70**:1621-8.
20. Conradi HJ, de Jonge P, Ormel J: **Differential treatment response in depressed patients with multiple prior episodes: CT versus the GP; Cognitive-behavioural therapy v. usual care in recurrent depression.** *Br J Psychiatry* 2008, **193**:505-6.
21. Bockting CLH: **The rhythm of depression: The course of recurrent depression and prevention of relapse using cognitive therapy.** *PhD thesis* Amsterdam; 2006.
22. Fava GA, Ruini C, Rafanelli C, Finos L, Conti S, Grandi S: **Six-year outcome of cognitive behavior therapy for prevention of recurrent depression.** *Am J Psychiatry* 2004, **161**:1872-1876.
23. Kuyken W, Byford S, Taylor RS, Watkins E, Holden E, White K, Barrett B, Byng R, Evans A, Mullan E, Teasdale JD: **Mindfulness-based cognitive therapy to prevent relapse in recurrent depression.** *J Consult Clin Psychol* 2008, **76**:966-978.
24. de Jong PJ, Pasman W, Kindt M, van den Hout MA: **A reaction time paradigm to assess (implicit) complaint-specific dysfunctional beliefs.** *Behaviour Res Therapy* 2001, **39**:101-113.
25. Beevers CG: **Cognitive vulnerability to depression: A dual process model.** *Clin Psychol Review* 2005, **25**:975-1002.
26. **Multidisciplinary Guidelines (Dutch).** *Guidelines Depression and Anxiety 2009* .
27. Morisky DE, Green LW, Levine DM: **Concurrent and predictive validity of a selfreported measure of medication adherence.** *Med Care* 1986, **24**:67-74.
28. Ma SH, Teasdale JD: **Mindfulness-based cognitive therapy for depression: Replication and exploration of differential relapse prevention effects.** *J Cons Clin Psychol* 2004, **72**:31-40.
29. Bockting CLH, Spinhoven Ph, Koeter MW, Wouters LF, Schene AH, the DELTA study group: **Prediction of recurrence in recurrent depression and the influence of consecutive episodes on vulnerability: a 2-year prospective study.** *J Clin Psychiatry* 2006, **67(5)**:747-755.
30. Spitzer RL, Williams JBW, Gibbon M, First MB: **The Structured Clinical Interview for DSM-III-R (SCID 1) history, rationale, and description.** *Arch Gen Psychiatry* 1992, **49(8)**:624-629.
31. Hamilton M: **A rating scale for depression.** *J Neurol Neurosurg Psychiatry* 1960, **23**:56-62.
32. Rush AJ, Gullion CM, Basco MR, Jarrett RB, Trivedi MH: **The Inventory of Depressive Symptomatology (IDS): psychometric properties.** *Psychol Med* 1996, **26**:477-86.
33. Kraaij V, De Wilde EJ: **Negative life events and depressive symptoms in the elderly: A life span perspective.** *Aging and Mental Health* 2001, **5**:84-91.
34. Franck E, De Raedt R, Barbez C, Rosseel Y: **Psychometric properties of the Dutch Rosenberg self-esteem scale.** *Pscyhologica Belgica* 2008, **48(1)**:25-35.
35. Akkerhuis GW, Kupka RW, van Groenestijn MAC, Nolen WA: *PDQ-4+ Vragenlijst voor persoonlijkheidskenmerken* Lisse: Swets & Zeitlinger; 1996.

36. van Hemert AM: *Lichamelijke klachten vragenlijst* Leids Universitair Medisch Centrum; 2003.
37. Vingerhoets AJJM, van Tilburg MAL: *Everyday Problem Checklist (EPCL)* Lisse: Swets & Zeitlinger B.V; 1994.
38. Angst J, Adolfsson R, Benazzi F, Gamma A, Hantouche E, Meyer TD, Skeppar P, Vieta E, Scott J: **Towards the self-assessment tool for hypomanic symptoms in outpatients.** *J Affective Disorders* 2005, **88**:217-233.
39. Hakkaart-van Roijen L, van Straten A, Donker M, Tiemens B: *Handleiding Trimbos/iMTA questionnaire for Costs associated with Psychiatric illness (TiC-P)* Instituut voor Medische Technology Assessment, Erasmus Universiteit Rotterdam; 2002.
40. EuroQol Group: **EuroQol: a new facility for the measurement of health-related quality of life.** *Health Policy* 1990, **16**:199-208.
41. Treynor W, Gonzalez R, Nolen-Hoeksema S: **Rumination reconsidered: A psychometric analysis.** *Cogn Therapy Res* 2003, **27**:247-259.
42. Weissman AN: **The Dysfunctional Attitude Scale.** *Academic dissertation* University of Pennsylvania (Dissertation Abstracts International: 40, 1389B-1390B); 1979.
43. Van der Does W: **Cognitive reactivity to sad mood: structure and validity of a new measure.** *Behav Res Ther* 2002, **40**:105-20.
44. Hayes SC, Strosahl KD, Wilson KG, Bissett RT, Pistorello J, Toarmino D, Polusny MA, Dykstra TA, Batten SV, Bergan J, Stewart SH, Zvolensky MJ, Eifert GH, Bond FW, Forsyth JP, Karekla M, Curry SM: **Measuring Experiential Avoidance: A preliminary test of a working model.** *Psychol Record* 2004, **54(4)**:553-578.
45. Schreurs PJG, Willige van den G, Brosschot JF, Tellegen B, Graus GMH: *Herziene handleiding De Utrechtse Coping Lijst: UCL. Omgaan met problemen en gebeurtenissen* Lisse: Swes & Zeitlinger; 1993.
46. First MB, Gibbon M, Spitzer RL, Williams JBW: **User Guide for the Structured Clinical Interview for DSM-IV Axis 1 Disorders.** Washington, DC: American Psychiatric Association; 1996.
47. Greenwald AG, McGhee DE, Schwartz JLK: **Measuring individual differences in implicit cognition. The Implicit Association Test.** *J Personality Social Psychol* 1998, **74**:1464-1480.
48. Koster EHW, De Raedt R, Verschuere B, Tibboel H, de Jong PJ: **Enhanced emotioninduced attentional blink for negative information in dysphoria.** *Depression and Anxiety* 2009, **26**:16-22.
49. Oostenbrink JB, Koopmanschap MA, Rutten FFH: *Manual for cost studies, methods and standard prices for economic evaluation in health care [In Dutch: Handleiding voor kostenonderzoek, methoden en richtlijnprijzen voor economische evaluaties in de gezondheidszorg. Geactualiseerde versie]* Amstelveen: Health Care Insurance Council; 2004.

Pre-publication history
The pre-publication history for this paper can be accessed here:
http://www.biomedcentral.com/1471-244X/11/8/prepub

doi:10.1186/1471-244X-11-8
Cite this article as: Bockting *et al.*: Disrupting the rhythm of depression: design and protocol of a randomized controlled trial on preventing relapse using brief cognitive therapy with or without antidepressants. *BMC Psychiatry* 2011 **11**:8.

Submit your next manuscript to BioMed Central and take full advantage of:

- **Convenient online submission**
- **Thorough peer review**
- **No space constraints or color figure charges**
- **Immediate publication on acceptance**
- **Inclusion in PubMed, CAS, Scopus and Google Scholar**
- **Research which is freely available for redistribution**

Submit your manuscript at
www.biomedcentral.com/submit

BioMed Central
